# A New Deep Learning-Based Method for Automated Identification of Thoracic Lymph Node Stations in Endobronchial Ultrasound (EBUS): A Proof-of-Concept Study

**DOI:** 10.3390/jimaging11010010

**Published:** 2025-01-05

**Authors:** Øyvind Ervik, Mia Rødde, Erlend Fagertun Hofstad, Ingrid Tveten, Thomas Langø, Håkon O. Leira, Tore Amundsen, Hanne Sorger

**Affiliations:** 1 Clinic of Medicine, Nord-Trøndelag Hospital Trust, Levanger Hospital, 7601 Levanger, Norway; hanne.sorger@ntnu.no; 2 Department of Circulation and Medical Imaging, Faculty of Medicine and Health Sciences, Norwegian University of Science and Technology, 7030 Trondheim, Norway; hakon.o.leira@ntnu.no (H.O.L.); tore.amundsen@ntnu.no (T.A.); 3 Department of Health Research, SINTEF Digital, 7034 Trondheim, Norway; mia.rodde@ntnu.no (M.R.); erlend.hofstad@sintef.no (E.F.H.); ingrid.tveten@sintef.no (I.T.); thomas.lango@sintef.no (T.L.); 4 National Research Center for Minimally Invasive and Image-Guided Diagnostics and Therapy, St. Olavs Hospital, 7030 Trondheim, Norway; 5 Department of Thoracic Medicine, St. Olavs Hospital, Trondheim University Hospital, 7030 Trondheim, Norway

**Keywords:** endobronchial ultrasound, AI-augmented EBUS, deep learning, deep neural networks, lymph node classification, lymph node staging

## Abstract

Endobronchial ultrasound-guided transbronchial needle aspiration (EBUS-TBNA) is a cornerstone in minimally invasive thoracic lymph node sampling. In lung cancer staging, precise assessment of lymph node position is crucial for clinical decision-making. This study aimed to demonstrate a new deep learning method to classify thoracic lymph nodes based on their anatomical location using EBUS images. Bronchoscopists labeled lymph node stations in real-time according to the Mountain Dressler nomenclature. EBUS images were then used to train and test a deep neural network (DNN) model, with intraoperative labels as ground truth. In total, 28,134 EBUS images were acquired from 56 patients. The model achieved an overall classification accuracy of 59.5 ± 5.2%. The highest precision, sensitivity, and F1 score were observed in station 4L, 77.6 ± 13.1%, 77.6 ± 15.4%, and 77.6 ± 15.4%, respectively. The lowest precision, sensitivity, and F1 score were observed in station 10L. The average processing and prediction time for a sequence of ten images was 0.65 ± 0.04 s, demonstrating the feasibility of real-time applications. In conclusion, the new DNN-based model could be used to classify lymph node stations from EBUS images. The method performance was promising with a potential for clinical use.

## 1. Introduction

Endobronchial ultrasound transbronchial needle aspiration (EBUS-TBNA) is the method of choice for minimally invasive assessment of thoracic lymph nodes [1,2]. In lung cancer, systematic mediastinal staging with cytology verification of nodal metastases impacts therapeutic decisions, particularly in the selection of patients for curative treatment [3,4].

**Figure 1:**
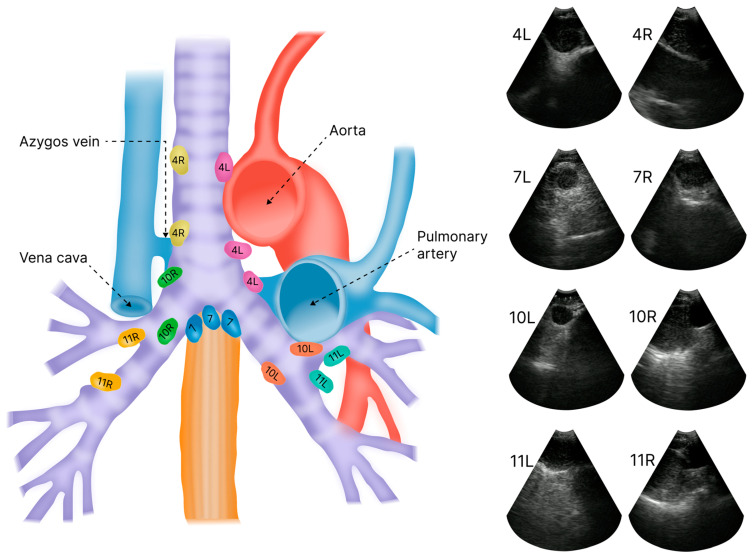
Regional mediastinal and hilar lymph node stations, including major blood vessels (**left**) and examples of EBUS images from the eight lymph node stations included in this study (**right**) [52].

Computed tomography (CT) and positron emission tomography with CT (PET-CT) can identify lymph nodes with suspected malignancy, indicating that tissue sampling with EBUS-TBNA is necessary for exact staging and decision-making [5,6]. The results from CT and PET-CT also form the basis for the bronchoscopist’s sampling strategy [2,7]. During the EBUS procedure, the initial positioning of the bronchoscope largely relies on direct endoluminal video visualization [8]. EBUS imaging is then used for real-time guidance to the target lymph node based on a continuous assessment of anatomical landmarks outside the airway wall [9]. The anatomical localization of lymph nodes is categorized into defined levels, named 1–14, according to the IASLC 8th edition [10,11]. The letters R or L indicate lateralization to the right or left side, respectively (Figure 1). The prognosis and treatment options for lung cancer depend on the presence and distribution of metastases in these lymph node levels [10,11]. EBUS-TBNA is considered to perform well in cytology confirmation of thoracic lymph node metastases. However, recent reports point to a 40% discrepancy between the estimated extent of mediastinal disease before and after lung cancer surgery [12,13,14,15,16]. This may indicate that today’s preoperative investigation with EBUS-TBNA is not yet precise enough and calls for improved mediastinal staging methods [15]. A major challenge is that EBUS-TBNA is operator-dependent [17,18,19,20,21]. Further, the lack of real-time access to CT and PET-CT images during sampling may hamper the operators’ ability to identify the exact lymph node of interest in ultrasound images. During video bronchoscopy, the video lens may become obscured by blood and mucus, making EBUS the main modality for further procedural guidance [22]. On the other hand, EBUS-guided localization of lymph nodes can be challenged by anatomical shifts due to breathing movements, tissue displacement exerted by the bronchoscope itself, and artifacts from cartilage and air between the probe and the target [23,24,25].

To make precise localization of thoracic lymph nodes with EBUS easier, advanced image-guiding techniques such as virtual bronchoscopy navigation (VBN) and electromagnetic tracking in EBUS have been suggested [26,27,28]. Based on CT images acquired before bronchoscopy, the CT view in VBN facilitates endobronchial navigation to the airway level closest to the predefined target, such as a lymph node. Electromagnetic tracking in EBUS is mainly used for research purposes [24,29]. The method principle is that an additional positioning and orientation tracking sensor attached to the bronchoscope or biopsy tool allows guided navigation in a 3D CT reconstructed volume that is registered to the patient’s lung [24,29].

The use of deep learning in the interpretation of EBUS images is relatively new and has been applied in the detection and segmentation of lymph nodes, as well as the classification of lymph nodes into benign or malignant [30,31,32,33,34,35,36,37,38,39,40,41,42]. Deep learning approaches based on convolutional neural networks (CNNs) have been used for image classification and object recognition tasks, significantly improving the interpretation of various medical imaging modalities, including ultrasound [43,44]. Furthermore, recurrent networks like the long short-term memory (LSTM) network have proven helpful in the interpretation of sequential data, such as text, audio, and video [45,46]. Combining convolutional and recurrent networks can improve performance in sequence prediction tasks with spatial inputs, like video-based challenges [47,48,49]. Therefore, a combined CNN and LSTM network could be well-suited for the automated interpreting of endoscopic ultrasound images. Yao et al. developed a system named EUS-MPS (endoscopic ultrasound–mediastinal position system) for the localization of mediastinal lymph nodes. This deep learning-based system was specifically designed for real-time recognition of lymph node stations during endoscopic ultrasound examinations of the upper gastrointestinal tract [50]. Similar approaches using deep learning to classify lymph node stations during ultrasound bronchoscopy have not been published. In this study, we aim to demonstrate a deep learning method that can classify thoracic lymph node stations during EBUS bronchoscopy automatically and in real-time.

## 2. Materials and Methods

### 2.1. Study Population

Patients referred to the Department of Pulmonology for EBUS-TBNA due to enlarged thoracic lymph nodes were prospectively enrolled without randomization. This study was approved by the Regional Committees for Medical and Health Sciences Research Ethics, Norway (identifier 240245, approval date 14 April 2021, and 588006, approval date 4 April 2023), and the Local Data Access Committee (identifier 2021/3210-19442/2021, approval date 21 June 2021, and 2023/1540-20710/2023, approval date 4 July 2023). Additionally, this study was registered at ClinicalTrials.gov (identifier NCT05739331, approval date 22 February 2023).

### 2.2. Preoperative Preparations

All patients completed a thorough clinical evaluation. This included pulmonary function tests and contrast-enhanced CT of the chest and abdomen. Patients with suspected lung cancer with the intention to cure also had PET-CT scans performed before EBUS-TBNA.

### 2.3. Intraoperative Workflow

**Figure 2:**
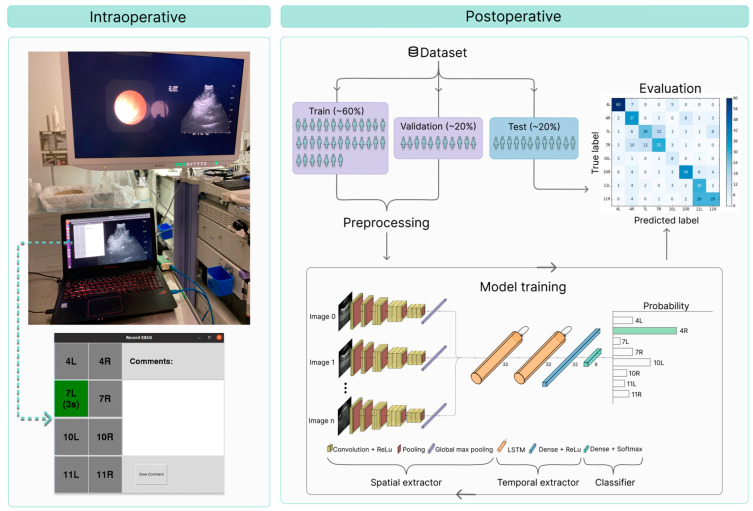
Intraoperative setup with the laptop computer used for video recording and labeling during EBUS-TBNA (**left**). A schematic diagram of the postoperative steps, including a patient-wise split of the dataset, preprocessing, and the training and evaluation of the DNN (**right**).

EBUS-TBNA was performed according to regional protocols using conscious sedation with midazolam and alfentanil. A flexible bronchoscope was first used for inspection, followed by a BF UC19OF ultrasound bronchoscope (Olympus, Tokyo, Japan) for the EBUS procedure. EBUS imaging was performed at a 10 MHz frequency and 40 mm image depth. Ultrasound videos were recorded from the EBUS processor (EU-ME2, Olympus, Tokyo, Japan) via a video grabber (AV.io, Epiphan Video, Palo Alto, CA, USA) to a laptop computer running custom software developed for this purpose. The bronchoscopist maneuvered the EBUS scope to obtain and record image sequences from lymph node stations 4L, 4R, 7L, 7R, 10L, 10R, 11L, and 11R, following the Mountain–Dresler system as specified in the 8th edition of the IASLC guidelines [7,10,11,51]. For this project, lymph node station 7 recordings were divided into 7R and 7L to distinguish imaging from the right and left sides of the main carina (Figure 1). We excluded lymph node stations 2, 5, 6, 8, and 12–14 from the study. Out of these, station 2 is the only station that can normally be accessed with the EBUS bronchoscope. However, sampling lymph nodes at this station presents technical challenges and is less likely to yield clear images and recordings. Therefore, we decided to begin with the simpler stations that are also part of the standard mapping procedures. During EBUS, the recordings were labeled with their corresponding lymph node stations on the laptop computer (Figure 2, left). All study procedures were conducted by two expert-level bronchoscopists, who had performed more than 500 EBUS procedures each.

### 2.4. Postoperative Image Processing

EBUS videos sampled at five images per second were used to train and test the DNN, using the bronchoscopist’s intraoperative lymph node station labels as ground truth. To prepare the dataset for machine learning, it was randomly divided at the patient level into training (60%), validation (20%), and test (20%) sets (Figure 2, right). During preprocessing, video segments labeled lymph node stations were split into sequences of ten EBUS images to extract temporal information. Furthermore, the images were cropped automatically to the smallest rectangular area containing the entire ultrasound sector and resized to 224 × 224 pixels. Data augmentation techniques such as gamma transform, non-linear mapping, Gaussian shadow, blurring, and rotation were applied to the sequences in the training set to prevent overfitting.

The model was trained using a combination of a two-dimensional CNN (MobileNetV3) and an LSTM network [45,53]. To enable real-time operation on lightweight GPUs for bronchoscopy, we have replaced the conventional flattening in CNNs with global max pooling, reducing parameters and potentially decreasing overfitting. As shown in Figure 2, the CNN extracts spatial features from each image in a sequence of *n* + 1 images. After global max pooling, the feature maps pass through two LSTM layers with 32 units each to extract temporal information from the sequence. Then, the classifier outputs a distribution that can be interpreted as probabilities of each of the eight lymph node stations. The model was trained in stateless mode, meaning that the hidden and cell states (memory) of the LSTM network are reset to zero at the start of each new batch. The training and later testing of the models were conducted using a computer equipped with an Intel(R) Xeon(R) Silver 4110 CPU and NVIDIA Quadro P5000 GPU. The model was trained for 500 epochs using a batch size of four and early stopping with the patience of 100 epochs. The categorical cross-entropy loss function was employed using the Adam optimizer with a learning rate of 0.0001 [54]. The model weights were saved at the epoch with the lowest validation loss.

The trained model was used to predict lymph node stations in both stateless and stateful modes on the test set. In stateful mode, memory is retained across batches. Therefore, by choosing a batch size of one, we enabled long-term memory across predictions by transferring information from one sequence to the next. It was included to improve the lymph node station predictions, particularly in videos with poor quality due to movement and artifacts.

To evaluate the model’s real-time potential in a clinical setting, the average processing and prediction time per sequence was estimated on the same computer used for training. It was calculated over ten runs, each consisting of 50 sequences with a length of ten images. To avoid initialization overhead, a warm-up run was performed before the beginning of the test.

## 3. Results

**Figure 3:**
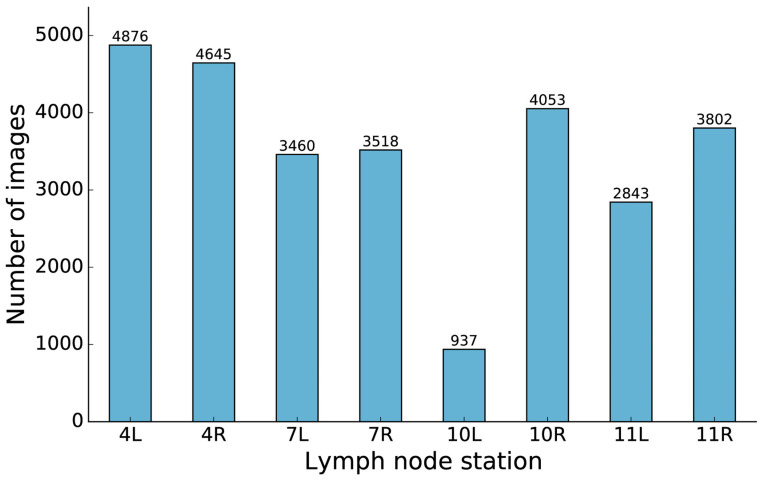
The number of images for each lymph node station indicates the balance of stations in the dataset.

A total of 28 134 EBUS images from 56 patients were collected. The distribution of images from the various lymph node stations is displayed in Figure 3.

Patient-based five-fold cross-validation was utilized to produce the results shown in Table 1. The table includes the mean value ± standard deviation (SD) for each lymph node station, presented for the classification model’s stateless and stateful modes. The model achieved an overall accuracy of 54.6 ± 5.6% for the stateless mode and 59.5 ± 5.2% for the stateful mode.

**Figure 4:**
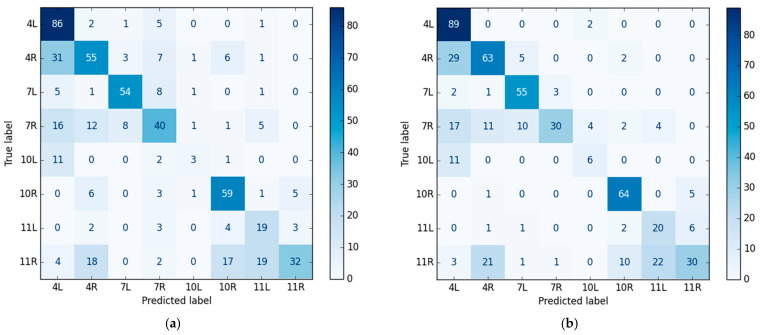
Confusion matrices for the classification model in stateless (**a**) and stateful modes (**b**) illustrate the results for the iteration that achieved the highest performance on its test set.

The matrices in Figure 4 show the number of correctly and incorrectly classified image sequences for each station based on a comparison of predicted and true labels. Figure 4a shows the confusion matrix of the iteration in the cross-validation that achieved the highest performance on its test set (in stateless mode). Figure 4b shows the confusion matrix on the same test set when temporal information from previous EBUS frames was incorporated (in stateful mode). The accuracy for this iteration was 61.4% and 66.9% for the stateless and stateful modes, respectively.

The average run time for processing and making a prediction based on a sequence of ten images was calculated to be 0.65 ± 0.04 s (mean ± SD).

## 4. Discussion

In this study, we demonstrate a new application for AI-enhanced EBUS guidance. We present a deep learning model that was able to classify the anatomical level of thoracic lymph nodes in near real-time using EBUS images as input. Based on EBUS videos from 56 patients, the overall model accuracy was 54.6 ± 5.6%. Compared to the capabilities of advanced deep learning models in other fields, the performance level for this application was relatively modest, mainly due to inferior results in one particular lymph node station. Further inclusion of patient data will probably improve model performance. Including temporal information from previous EBUS images raised the accuracy to 59.5 ± 5.2%, suggesting further potential for enhanced performance in poor-quality EBUS videos. The average run time for a station prediction was 0.65 ± 0.04 s, indicating that real-time implementation in the operating room should be possible.

As indicated in Table 1, our network performed well in all lymph node stations except for 10L. The study data contained limited images from 10L lymph nodes. This could be explained by random variation in our patient population. There is also an inherent difficulty locating the 10L station during EBUS bronchoscopy, a challenge that has been noted in previous EBUS-TBNA studies [40,55]. Expanding the dataset could improve model precision and sensitivity in the future. The model had difficulty distinguishing between stations 11R and 11L, as well as between 7R and 7L. This could be due to the similarity of anatomical landmarks, such as blood vessels, in ultrasound images from these regions. However, in a real-life setting, the distinction between the right and left side is usually intuitive for the bronchoscopist. A more important clinical point is that certain lymph node stations, such as 4R and 10R and 4R and 4L, are closely localized. It can be a real challenge to separate these with EBUS, yet it is crucial for correct treatment decisions in lung cancer. The network’s ability to distinguish between these stations is promising, underscoring its potential clinical usefulness.

We have not been able to identify other studies using deep learning to classify lymph node stations from EBUS images. Therefore, a direct comparison of model performance for this specific clinical application is not possible. However, Yao et al. have developed EUS-MPS, a deep learning-based system designed for the localization of lymph node stations during endoscopic ultrasound examinations of the upper gastrointestinal tract [50]. Compared to our results, they achieved a higher accuracy of 83.80% in video validation. Their work underpins the need for improved ultrasound guidance during endoscopic lymph node sampling and the importance of additional studies such as ours to explore deep learning for this purpose. There is still a need for dedicated deep learning studies for EBUS-TBNA since there are procedure-specific factors challenging EBUS imaging: First, the occurrence of artifacts from air and cartilage within the airways. Second, the constant movement of anatomical structures due to cough and respiration during instrumentation of the airways. Third, the short distance of just a few millimeters between mediastinal lymph nodes that belong to separate lymph node stations is a particular challenge during cancer staging. These factors may not be equally challenging during US imaging from the gastrointestinal tract. From a technical point of view, their study separates from ours since they trained three CNN models using ResNet-50, where the first two models select images of good quality to be included in the final prediction of the third model. We used all images as input to a combined CNN (MobileNetV3) and LSTM network. This can partly explain the lower accuracy in our study, along with the more challenging data outlined above. The advantage of our method is its ability to process image frames more quickly, generating new predictions every 650 ms per sequence as opposed to every 200 to 300 ms reported in the study by Yao’s group. Since each image sequence consists of 10 frames, this results in 65 ms per frame. Bronchoscopy guidance methods that are partly comparable to ours include electromagnetic tracking [24,29]. However, electromagnetic tracking is notably more resource-intensive, whereas our method is a software-only solution that can be incorporated into any EBUS system [56]. On the other hand, these methods are currently more robust since they do not rely on high-quality ultrasound images. Previous studies using deep learning to interpret EBUS images focus on a different clinical challenge: the distinction between benign and malignant lymph nodes [30,31,32,33,34,35,36,37,38,39,40,41,42]. Current clinical guidelines for lung cancer still recommend cytology samples to confirm or exclude lymph node metastases, highlighting the need for methods that improve the accuracy of TBNA sampling [1,2].

Our method improves the identification of thoracic lymph node stations during EBUS, especially in situations with reduced bronchoscope video vision or atypical anatomy. When a lymph node is visualized with the EBUS probe, the bronchoscopist can receive immediate feedback about its exact position inside the thoracic cavity. Combined with the knowledge of suspected metastatic sites from CT and PET-CT, this could aid the decision of whether TBNA sampling should or should not be performed.

In our study, retaining the memory between image sequences was useful to distinguish between lymph node stations. It reduced switching between different predictions within videos recorded from the same anatomical area. This feature strengthens the model’s ability to handle ultrasound images of various qualities, a key advantage during EBUS procedures where air artifacts are frequent. We selected a sequence length of 10 images to provide sufficient temporal context for accurate predictions while trying to minimize complexity. In the context of EBUS videos, this corresponds to updates approximately every two seconds, enabling timely feedback for the bronchoscopist.

Although the study population included a limited number of patients, the resulting dataset contained a high total number of EBUS images used to develop our deep learning model. All the study participants presented with enlarged thoracic lymph nodes; no other selection was made based on specific diagnoses. The dataset consisted of EBUS videos, including images with artifacts due to air, cartilage, and respiratory movements and images labeled as ‘low quality’ by the bronchoscopist. This approach ensured a thorough evaluation of the model’s robustness under suboptimal conditions. The variable image quality in our dataset could have influenced the observed model accuracy. On the other hand, our study results should reflect real-life conditions.

The majority of study data was collected by two bronchoscopists from the same hospital. The experts’ experience, knowledge, and image interpretation skills were pivotal as the ground truth in model training and could have introduced experimental error. Additionally, patient movements, such as breathing or coughing, could have caused the EBUS probe and ultrasound image to switch inadvertently between stations, potentially leading to incorrect image labels [57]. These challenges could be particularly relevant in anatomically adjacent stations, like 4R and 10R, where even slight positional shifts could lead to classification error.

There are some study limitations. First, the size of the dataset could have affected the robustness and generalizability of our results. Second, the study involved only two EBUS experts, which could have introduced bias caused by a site-specific EBUS-TBNA workflow or a particular EBUS training program. Third, due to the single-center design, the model has not yet been validated on data collected with different medical technology equipment. Lastly, the lack of external validation limits the generalizability of the findings to broader populations or settings since our results may be influenced by the specific characteristics of the analyzed data. In this study, validation on previously unseen data was not possible since we did not have access to other datasets. There is a need for future studies that test our method on external data to confirm the reproducibility and robustness of the results. We were not able to find a publicly available dataset containing EBUS images. Thus, we had to design and conduct a clinical study from the start, which limited the achievable size of the dataset.

To allow interobserver cross-checking, video recordings could also be incorporated, along with ultrasound. Involving multiple study sites, different EBUS systems, and bronchoscopists at various levels of expertise could reduce the risk of operator- or institution-specific bias and make it possible to evaluate interobserver variability. Increasing the number of patients and the total number of training images could improve model performance in the future.

In lung cancer, the results from lymph node sampling with EBUS-TBNA are used to stratify patients between surgical or non-surgical, curative, or palliative treatment strategies. It is essential to know exactly which lymph node stations contain cancer metastases and which do not to make correct therapeutic decisions. In this context, the study limitations could impact clinical outcomes by leading to overestimation or underestimation of cancer spread in cases where the model’s predictions changed the estimated stage of disease. This could lead to unnecessary surgery and risk in patients with advanced disease or prevent patients with early-stage disease from receiving curative treatment, reducing their chances for long-term survival. Addressing the limitations of our study is, therefore, a necessary step toward the clinical implementation of AI-augmented EBUS-TBNA.

This proof-of-concept study represents a clear advancement within deep learning-assisted medical ultrasound, introducing a new clinical application. We provide new insight into the potential of deep learning for real-time analysis of endobronchial EBUS images, an area that has been subject to relatively few studies compared to, for example, cardiac ultrasound. The current study resulted in a new and unique dataset that has never been used for AI research before, providing a valuable starting point for future studies exploring deep learning analyses of EBUS. Our innovative methodology employs advanced model architectures specifically adapted to address the unique challenges of EBUS imaging, providing precedence for future research.

Although the model’s accuracy must be improved before we apply it in regular clinical use, it already performs well for several lymph node stations such as 4R and 10R, in which a correct classification is clinically important since, in some patients, the verification of lymph node metastases to 4R could rule out surgery. The presented results could, therefore, be considered promising. Today’s EBUS without AI augmentation is highly operator-dependent, and there are already inherent challenges in the EBUS classification of certain lymph node stations [17,18,19,20,21]. Thus, advances to improve standardization and support of ultrasound-guided classification should be of clinical interest. To ensure further clinical value of our deep learning method, we are conducting explainability analyses to better understand what factors influence the model’s predictions. In future studies, we intend to incorporate visualization techniques, such as Gradient-weighted Class Activation Mapping (Grad-CAM), to highlight key regions in the EBUS images used by the model to predict lymph node stations [58]. Additionally, future research could include the optimization of hyperparameters to achieve faster learning and greater accuracy.

In a previous study, we used deep learning in the segmentation of important anatomical structures in EBUS images [36]. This real-time automatic segmentation helps to locate and detect anatomical landmarks, which is especially useful during EBUS-guided sampling of thoracic lymph nodes. By combining deep learning methods to classify thoracic lymph nodes based on their location and real-time segmentation of critical landmarks, such as lymph nodes and blood vessels, our approach aims to enhance the navigation of the EBUS bronchoscope for optimal TBNA sampling. We plan to test this method in the bronchoscopy suite during patient procedures, where both the bronchoscopist and the DNN will be blinded to each other’s results.

A major clinical benefit of the proposed method is that lymph node station localization relies solely on EBUS images. Thus, the method could be particularly useful in reduced or poor-quality video vision procedures. In real-life situations, we believe that using deep learning-enhanced EBUS guidance can help speed up anatomical orientation, improve lung cancer staging, and provide more accurate staging assessments before surgery. This advancement could reduce procedure times, especially in EBUS-TBNAs that involve multiple lymph node samples. This method may strengthen the performance of less experienced bronchoscopists and enhance endoscopy training.

## 5. Conclusions

This study successfully demonstrated a new deep learning-based method for classifying lymph node stations during EBUS. The model performance was promising, with potential for clinical use during EBUS-TBNA and lung cancer staging. The findings suggest that deep learning-enhanced EBUS guidance has the potential to improve EBUS-TBNA procedures using a specialized software-only solution, offering numerous possibilities for future advancements.

## Figures and Tables

**Table 1:** Precision (positive predictive value) and sensitivity (recall) results for the classification model for each lymph node station in the stateless and stateful modes.

Metric	Precision (%)		Sensitivity (%)		F1-Score (%)	
Model	Stateless	Stateful	Stateless	Stateful	Stateless	Stateful
4L	70.8 ± 12.1	77.6 ± 13.1	70.0 ± 15.3	77.6 ± 15.4	69.3 ± 10.6	76.2 ± 10.4
4R	59.5 ± 6.2	65.2 ± 5.1	56.8 ± 7.6	63.5 ± 9.0	57.3 ± 2.1	63.7 ± 3.0
7L	57.7 ± 15.7	57.6 ± 13.9	60.6 ± 12.3	70.7 ± 13.5	57.4 ± 11.2	62.0 ± 11.5
7R	47.3 ± 9.9	61.4 ± 16.1	52.5 ± 4.8	50.0 ± 13.8	49.0 ± 5.5	53.4 ± 9.8
10L	29.8 ± 27.2	32.3 ± 29.1	17.8 ± 21.0	26.3 ± 29.2	18.4 ± 16.5	24.0 ± 20.7
10R	62.1 ± 7.3	68.2 ± 6.6	61.8 ± 12.0	68.7 ± 19.1	61.7 ± 9.3	67.2 ± 13.4
11L	39.6 ± 6.3	40.3 ± 6.0	50.1 ± 14.7	51.5 ± 14.2	43.2 ± 8.0	44.2 ± 7.8
11R	53.0 ± 18.0	54.4 ± 18.3	36.4 ± 7.2	38.9 ± 10.3	41.7 ± 8.1	44.1 ± 10.6

Data are represented as mean ± SD.

## Data Availability

The datasets generated and/or analyzed in the current study are not publicly available since they are part of an ongoing study. Data might be available upon reasonable request on a mutually collaborative basis. Please contact the first author, Øyvind Ervik.
